# Vesicular Ganglioside GM1 From Breast Tumor Cells Stimulated Epithelial-to-Mesenchymal Transition of Recipient MCF-10A Cells

**DOI:** 10.3389/fonc.2022.837930

**Published:** 2022-04-26

**Authors:** Qilong Ma, Dinghao Zhuo, Feng Guan, Xiang Li, Xiaomin Yang, Zengqi Tan

**Affiliations:** ^1^Joint International Research Laboratory of Glycobiology and Medicinal Chemistry, College of Life Science, Northwest University, Xi’an, China; ^2^Women's Hospital, and Institute of Genetics, Zhejiang University School of Medicine, Hangzhou, China; ^3^School of Medicine, Northwest University, Xi’an, China; ^4^Department of Breast Surgery, The First Affiliated Hospital of Xi’an Jiaotong University, Xi’an, China; ^5^Department of Breast Surgery, Tumor Hospital of Shaanxi Province, Xi’an, China

**Keywords:** GM1, small extracellular vesicle, B3GALT4, epithelial–mesenchymal transition, breast cancer

## Abstract

Small extracellular vesicles (sEVs) are a type of membrane structure secreted by cells, which are involved in physiological and pathological processes by participating in intercellular communication. Glycosphingolipids (GSLs) are enriched in sEV and can be delivered to recipient cells. In this study, we found that overexpression of B3GALT4, the glycosyltransferase responsible for ganglioside GM1 synthesis, can induce the epithelial–mesenchymal transition (EMT) process in MCF-10A cells. Moreover, GM1 was verified to be presented on sEV from breast cancer cells. Overexpression of B3GALT4 resulted in elevated vesicular GM1 levels and increased sEV secretion in breast cancer cells. Proteomic analysis revealed that eleven sEV secretion-related proteins were differentially expressed, which might contribute to the altered sEV secretion. Of the identified proteins, 15 oncogenic differentially expressed proteins were documented to be presented in sEV. With the treatment of GM1-enriched sEV from breast cancer cells, the EMT process was induced in recipient non-tumorigenic epithelial MCF-10A cells. Our findings demonstrated that GM1-enriched sEVs derived from breast cancer cells induced the EMT process of recipient cells, which might provide essential information on the biological function of vesicular GM1.

## Introduction

Small extracellular vesicle (sEV), one type of membranous microvesicles (diameter 30–100 nm), is secreted by all cell types, and found in various body fluids (1, 2). sEV can be loaded with different cargos, including proteins, nucleic acids, and lipids derived from parental cells (3). sEV is able to mediate local and systemic cellular communication *via* horizontal transfer of cargos to recipient cells, and influence the environment in both physiological and pathological processes (4–7). Specifically, tumor-derived sEV can be internalized and conferred malignant characteristics in recipient cells, such as evading growth suppressors, promoting angiogenesis, exerting immunomodulatory effects, enhancing migration and invasion, and inducing epithelial–mesenchymal transition (EMT) (8–11).

EMT is an important feature of tumorigenesis processes and also one hallmark of tumor cell invasion and metastasis (12–14). EMT refers to the process in which epithelial cells lose their epithelial phenotypic characteristics such as polarity and intercellular adhesion connections, and transform into mesenchymal cells with high migration and invasion ability (15, 16). Tumor-derived sEV can deliver cargos including TGF-β, β-catenin, and various micro-RNAs, which enhance the invasion and migratory ability and induce EMT process in recipient cells (17).

Similar to cell membrane, sEV surfaces are also abundantly covered by glycoconjugates including glycosphingolipids (GSLs), proteoglycans, and glycoproteins (18). GSLs are key components of eukaryotic cellular membranes and are clustered with cholesterol in lipid rafts (also termed GSLs/cholesterol-enriched membrane microdomains) (19). Suppressed synthesis of GSLs could affect the composition of sEV released from human prostate cancer PC-3 cells (20). Gangliosides, a major group of GSLs bearing one or more sialic acid residues, are known as mediators of cell adhesion and modulators of signal transduction, in addition to their classically known functions as antigens and receptors (21). Ganglioside GD3-enriched EVs could stimulate the migration of melanocyte cells (22). Ganglioside GM1, one typical lipid raft component, was localized in detergent-resistant fractions isolated from Daudi-secreted sEV, and might participate in vesicle formation and structure (23).

GM1 is a sialotetraosylceramide consisting of a branched pentasaccharide made up of one sialyl residue, two galactose (Gal) residues, one N-acetylgalactosamine (GalNAc) residue, and one glucose (Glc) residue at the reducing end attached to ceramide *via* a β-linkage. β-1,3-galactosyltransferase 4 (B3GALT4), which catalyzes the transfer of Gal from UDP-Gal to GalNAc in ganglioside, is one of the key enzymes responsible for GM1 synthesis (24). It is well known that GM1 plays a specific role in neuronal differentiation and protection, *via* interacting with various plasma membrane proteins and further triggering the activation of intracellular biochemical pathways (25). GM1 also shows important functions of regulating cell proliferation, movement, and adhesion (26).

In our previous study, we found that GM1 levels were increased in high-density breast normal and cancer epithelial cells, and exogenous addition of GM1 to high-density cells could clearly promote the contact inhibition of growth by deactivation of EGFR signaling (27). However, the effect of GM1 on the biological function of sEV is not well elucidated. In this study, we validated the presence of GM1 on sEV derived from breast cancer cells, and explored the mechanism underlying the increased sEV secretion in B3GALT4-overexpressed cells and the vesicular GM1 induced EMT process in recipient cells by proteomic analysis. Moreover, we described here the manner in which GM1 modulated the function of sEV and behaviors of recipient cells.

## Materials and Methods

### Cell Culture

Human breast cancer cell lines (MDA-MB-231 and MCF-7) and human normal mammary epithelial cell line MCF-10A were purchased from the Cell Bank of the Chinese Academy of Sciences (Shanghai, China). B3GALT4-overexpressed or -silenced MDA-MB-231 and MCF-10A cells were previously established in our lab (27). Transfected, parental MDA-MB-231 and MCF-7 cells were cultured in DMEM (Biological Industries, Beit Haemek, Israel) containing 10% fetal bovine serum (Biological Industries) and 1% penicillin/streptomycin (Beyotime, Haimen, Jiangsu, China). Transfected and parental MCF-10A cells were cultured in DMEM/F12 (Gibco, Thermo Fisher Scientific; San Jose, CA, USA) supplemented with 5% horse serum (Thermo Fisher Scientific), hydrocortisone, epidermal growth factor (EGF, Peprotech; Rocky Hill, NJ, USA), cholera toxin, recombinant human insulin (Sigma-Aldrich; St. Louis, MO, USA), and 1% penicillin/streptomycin. All cells were cultured at 37°C in 5% CO_2_ atmosphere.

### Isolation of sEV

sEV was isolated from culture supernatant as previously described (28). Cells were cultured in exosome-free DMEM medium for 48 h. The conditioned medium was collected and centrifuged at 500 g for 10 min, 2,000 *g* for 20 min, and 11,000 *g* for 30 min at 4°C, respectively. The supernatant was collected and subjected to ultracentrifugation at 110,000 *g* for 70 min (Optima XE-100; Beckman Coulter Life Sciences; Indianapolis, IN, USA). sEV pellets were collected and ultracentrifuged at 110,000 *g* for 70 min again. sEV pellets were resuspended in 100 μl of PBS and stored at −80°C.

### OptiPrep Density Gradient Centrifugation

sEV was separated by modified OptiPrep density gradient centrifugation (29). The OptiPrep stock solution [60% (w/v) aqueous iodixanol, Axis-Shield PoC; AS; Oslo, Norway] was diluted to 40%, 20%, 10%, and 5% (w/v), and each component was continuously loaded into 14 × 89 mm Ultra-Clear tubes (Beckman Coulter). sEVs obtained from ultracentrifugation were carefully added to the top layer. Tubes were placed in sw-41 rotor (Beckman Coulter) and centrifuged at 100,000 *g* for 18 h. Twelve fractions were collected. Each fraction was diluted in PBS, ultracentrifuged at 110,000 *g* for 3 h, and resuspended with PBS.

### Transmission Electron Microscopy (TEM)

TEM assay of sEV was modified based on a previously described procedure (30, 31). Purified sEV was applied to carbon-coated 400-mesh grids (Electron Microscopy Sciences; Fort Washington, PA, USA) for 5 min, washed with PBS, and stained with 1% uranyl acetate (Sigma-Aldrich) for 2 min. The excess liquid was carefully absorbed with filter paper. sEV was observed and photographed by TEM (model H-7650; Hitachi; Tokyo, Japan) at 80 kV.

### Western Blot

Cells were lysed with RIPA buffer containing 1% protease inhibitor cocktail, 50 mmol/L Tris-HCl, 150 mmol/L NaCl, 1% sodium deoxycholate, 1% Triton X-100, and 0.1% SDS. Cell lysates were centrifuged, and supernatants were collected. Proteins were loaded and separated by SDS-PAGE. After electrophoresis, proteins were transferred onto polyvinylidene fluoride (PVDF) membranes (Bio-Rad; Hercules, CA, USA). Membranes were blocked with 3% bovine serum albumin (BSA, Beyotime) in TBST for 1 h at 37°C, and probed with primary antibodies overnight at 4°C, and incubated with appropriate HRP-conjugated secondary antibodies. Bands were visualized by enhanced chemiluminescence (ECL; Vazyme Biotech, Nanjing, China). The commercially available antibodies used in this study were listed as follows: primary antibodies against CD63 (#ab134045), TSG101 (#ab83) (Abcam; Cambridge Cambridgeshire, UK), Alix (#2171S), Calnexin (#2679S) (Cell Signaling Technology, Danvers, MA, USA), E-cadherin (#610181) (Becton, Dickinson and Company; Sparks, MD, USA), CD81 (#sc-23962), fibronectin (#sc-271098), vimentin (#sc-6260) (Santa Cruz Biotechnology; Santa Cruz, CA, USA), and GAPDH (#G9545, Sigma-Aldrich).

### Flow Cytometry Assay

Cells were digested with trypsin, washed twice with PBS, fixed with 4% fresh paraformaldehyde, blocked with 1% BSA in PBS, and incubated with fluorescein isothiocyanate-conjugated Cholera Toxin B subunit (CTB-FITC, #C1655, Sigma-Aldrich) at 37°C for 30 min. Fluorescence signals were detected and analyzed using flow cytometry (ACEA Biosciences, San Diego, CA, USA).

### Transwell Assay

Cells were cultured until they reached 70%–80% confluence. Cells were digested with trypsin, washed with PBS, resuspended in serum-free medium, and inoculated in the upper chamber of a 0.8-μm transwell filter (Corning; Cambridge, MA, USA). Complete medium was added into the lower chamber. After 24-h culture, migrated cells were dyed with 0.1% crystal violet and photographed under a microscope.

### Hanging Drop Assay

To elucidate the cell–cell adhesion, the hanging drop aggregation was performed as previously described (32). Briefly, cells that reached a confluency of 80% were detached by trypsin and resuspended in complete medium. The single-cell suspension of 2×10^4^ cells in 30 μl of medium was suspended as a hanging drop from the lid of a 6-well plate and allowed to aggregate overnight at 37°C in 5% CO_2_. Cells were subjected to shear force by passing through a 200-μl pipette tip 10 times, and photographed after mechanical stress.

### Cell Adhesion Assay

To elucidate the adhesion strength between cells and extracellular matrix (ECM), the cell adhesion assay was performed as previously described (33). Briefly, the single-cell suspension of 5×10^4^ cells in 500 μl of medium was inoculated in a 48-well plate, and incubated for 4 h at 37°C in 5% CO_2_. Attached cells were rinsed with PBS, stained with 0.1% crystal violet, and photographed under a microscope.

### Glycosphingolipid Extraction and Analysis

GSLs were extracted and analyzed as previously described (34, 35). Cells (pellets harvested by centrifugation) were extracted twice with 2 ml of isopropanol/hexane/water (55:25:20, v/v/v) with vortexing and sonication, and centrifuged. Extracts were evaporated to dry under nitrogen stream. The residue was incubated with 2 ml of 0.1 mol/L NaOH in methanol at 40°C for 2 h to hydrolyze phospholipids, neutralized with 1 mol/L HCl, and added with 2 ml of hexane. The upper layer was removed from the lower layer. GSLs in the lower phase were dried, solubilized in 1 ml of distilled water, and desalted with SepPak C18 cartridge (Waters Corporation, Milford, MA, USA). Glycan components of GSLs were released by EGCase treatment (36) and subjected to LC-MS analysis.

### Analysis of sEV Uptake

sEV from breast cancer cells was incubated with CFSE (#21888, Sigma-Aldrich) at 37°C for 30 min in the dark (8), purified by ultracentrifugation at 110,000 *g* for 70 min to remove excess CFSE, and incubated with MCF-10A cells at 37°C for 30 min. Images were acquired by laser confocal microscopy (model TCS SP8; Leica; Weztlar, Germany). The sEV uptake was quantified by calculating the integrated optical density of the green signal in the field using Image Pro Plus software.

### Detection of Vesicular GM1 Transfer

GM1 labeling was performed as previously described (37, 38). Briefly, sEV from MDA-MB-231 cells was incubated with biotinylated CTB (CTB-Biotin, #C9972, Sigma-Aldrich) on ice for 1 h, purified by ultracentrifugation at 110,000 *g* for 70 min to remove excess CTB-Biotin, and incubated with MCF-10A at 37°C for 6 h. Western blot was performed to explore the transfer of biotin-labeled vesicular GM1 in recipient cells.

### Statistical Analysis

All experiments were performed in triplicate and data were analyzed by GraphPad Prism (version 9.0). The experimental data between two groups were analyzed using two-tailed Student’s *t*-test. Differences with *p* < 0.05 were considered as statistically significant. *p < 0.05, **p < 0.01, ***p < 0.001.

## Results

### GM1 Affects EMT Process in MCF-10A Cells

**Figure 1:**
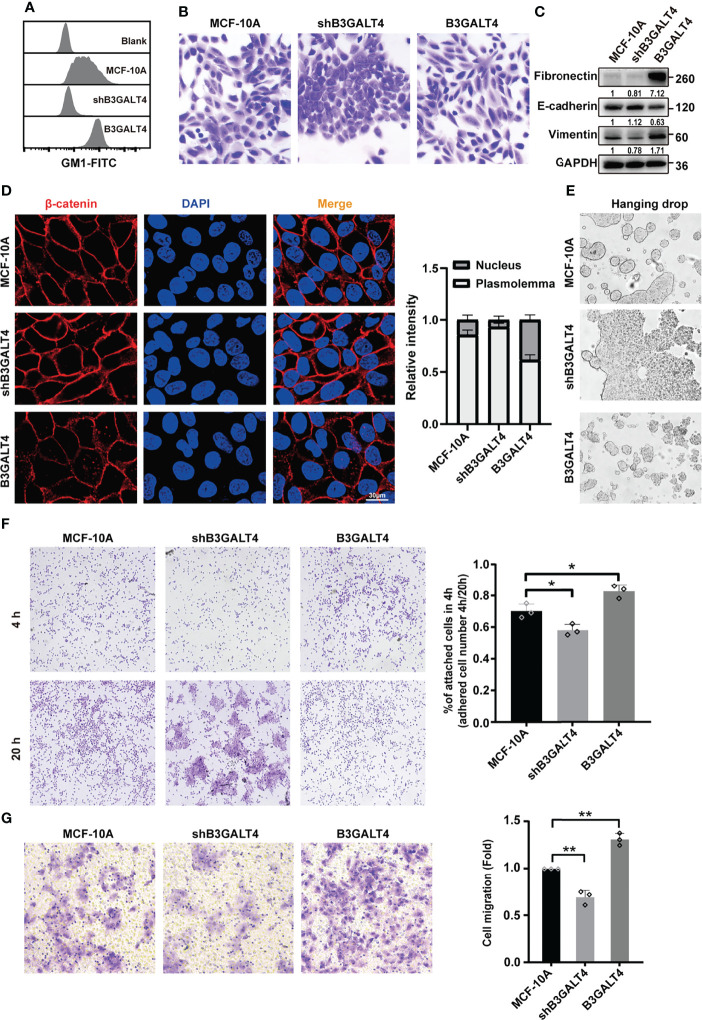
GM1 induces EMT process in MCF-10A cells. **(A)** Expression of GM1 in parental, B3GALT4-transfected and -silenced MCF-10A cells (termed as B3GALT4 or shB3GALT4), evaluated by flow cytometry. Blank indicates unstained MCF-10A cells. MCF-10A, shB3GALT4, and B3GALT4 indicate parental, B3GALT4-silenced, and B3GALT4-overexpressed cells stained with CTB-Biotin. **(B)** Morphology of parental, B3GALT4-transfected and -silenced MCF-10A cells. **(C)** Expression of fibronectin, E-cadherin, and vimentin of cells evaluated by Western blot. **(D)** Expression and localization of β-catenin evaluated by immunofluorescence assay. **(E)** Cell–cell adhesion of parental, B3GALT4-transfected and -silenced MCF-10A cells evaluated by hanging drop aggregation assays. **(F)** The cell-matrix adhesion of cells evaluated by cell adhesion assay. **(G)** The migratory ability of parental, B3GALT4-transfected and -silenced MCF-10A cells evaluated by transwell assay. The above experiments were performed in triplicate wells and independently conducted three times. *p < 0.05, **p < 0.01.

GSLs are integral components of mammalian cell membranes, and play essential roles in cell adhesion and signaling activation. We have previously demonstrated that GM1 can regulate cell proliferation in human mammary epithelial cells under high-density conditions *via* EGFR signaling (27). Effects of GM1 on the EMT process and its underlying mechanism need further study. In the present study, immunohistochemical analysis showed that the expression of B3GALT4 was higher in breast cancer tissues than in paired normal tissues (**Figure S1A**). To investigate the biological function of GM1, glycosyltransferase B3GALT4 (GM1 synthase) gene was overexpressed and silenced in MCF-10A cells, respectively. High level of GM1 in B3GALT4-overexpressed cells and low level of GM1 in B3GALT4-silenced cells were confirmed by flow cytometry (**Figure 1A**), immunofluorescence (**Figure S1B**), and Western blot (**Figure S1C**). B3GALT4-overexpressed cells presented a spindle-like morphology and loose cell-to-cell adhesion. B3GALT4-silenced cells were more rounded and tightly adhered (**Figure 1B**). The EMT process of MCF-10A was facilitated by high level of GM1, as evidenced by E-cadherin downregulation, and fibronectin and vimentin upregulation, and was suppressed by low level of GM1 (**Figures 1C**, **S1C**). β-catenin is a component of cell adhesion complex, and nuclear translocation of β-catenin activates the related transcription factors, transcribes Wnt target genes, and mediates the occurrence of EMT (39). Increased nuclear expression of β-catenin is the hallmark of the activation of Wnt/β-catenin signaling pathway. Immunofluorescence analysis showed that the β-catenin was translocated from the cytoplasm to the nucleus by high GM1 level (**Figure 1D**).

Cell–cell adhesion was suppressed by B3GALT4 overexpression in MCF-10A cells, as evidenced by sensitivity to relatively strong mechanical stress using cell adhesion assay, and was enhanced by B3GALT4 knockdown (**Figure 1E**). Furthermore, cell-matrix adhesion was significantly enhanced by B3GALT4 overexpression, but suppressed by B3GALT4 knockdown (**Figure 1F**). Similarly, the migratory ability of MCF-10A was increased by B3GALT4 overexpression, but reduced by B3GALT4 knockdown (**Figure 1G**). Together, these results suggested that high GM1 level facilitated the EMT process, accompanied by elevated migratory ability in MCF-10A.

We also investigated the effect of GM1 on the TGF-β-induced EMT process. TGF-β-induced EMT was facilitated by endogenous B3GALT4 overexpression with increased expression of mesenchymal marker and decreased epithelial marker (**Figures S2A, B**), but not by B3GALT4 silencing. Besides GM1, B3GALT4 is also responsible for the synthesis of GD1b and GT1b. To examine the ganglioside expression pattern in B3GALT4-transfected cell, glycan components of GSLs from parental and B3GALT4-silenced MCF-10A cells were profiled by mass spectrometry (**Figure S2C**). We found that a total of 9 distinct GSLs were identified in both cell lines. Glycan structures and relative intensity of identified GSLs were summarized in **Table S1**. These 9 GSLs, including GM1, were all significantly downregulated in B3GALT4-silenced MCF-10A cells compared to parental cells (**Figure S2D**). To investigate the biological function of GM1 other than other GSLs produced by B3GALT4, MCF10A cells were treated with TGF-β, one typical EMT inducer, and fed with exogenous GM1. The mesenchymal marker expression was increased, and epithelial marker expression was decreased by exogenous GM1 treatment in treated MCF10A cells (**Figure S2E**). These data showed that GM1 of 9 decreased GSLs is mainly responsible for the EMT process.

### Secretions From Donor Cells Induce the EMT Process of MCF-10A

**Figure 2:**
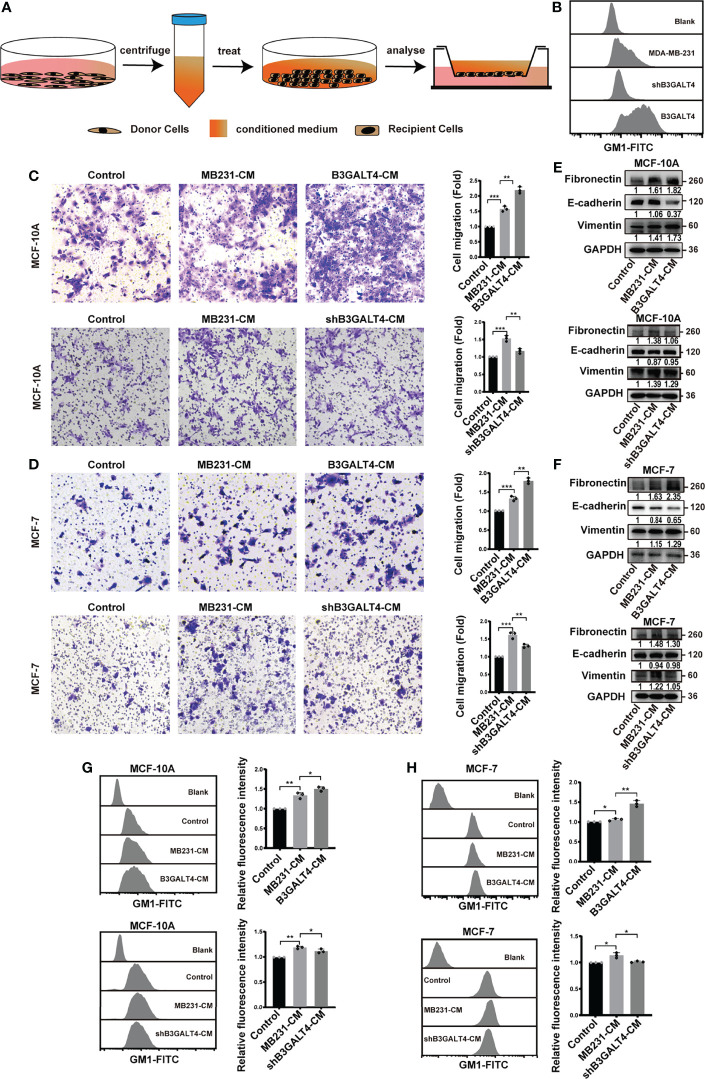
Conditioned medium of B3GALT4-overexpressed cells affect EMT process of MCF-10A cells and MCF-7. **(A)** Schematic model of conditioned medium treatment. Conditioned medium was collected as described in Materials and Methods. Recipient cells were incubated with CM, and then subjected to transwell assay, Western blot, and flow cytometry analysis. **(B)** GM1 levels in parental, B3GALT4-overexpressed and -silenced MDA-MB-231 cells were determined by flow cytometry. B3GALT4, B3GALT4-overexpressed MDA-MB-231 cells, shB3GALT4, B3GALT4-silenced cells. Blank indicates unstained MDA-MB-231 cells. MDA-MB-231, shB3GALT4, and B3GALT4 indicate parental, B3GALT4-silenced, and B3GALT4-overexpressed MDA-MB-231 cells stained with CTB-Biotin. **(C, D)** The migratory ability of MCF-10A **(C)** and MCF-7 cells **(D)** treated with B3GALT4-CM and shB3GALT4-CM. **(E, F)** The expression of fibronectin, E-cadherin, and vimentin in MCF-10A **(E)** and MCF-7 cells **(F)** treated with B3GALT4-CM and shB3GALT4-CM, evaluated by Western blot. **(G, H)** GM1 levels in MCF-10A **(G)** and MCF-7 cells **(H)** treated with B3GALT4-CM and shB3GALT4-CM, evaluated by flow cytometry. Blank indicates unstained MCF-10A or MCF-7 cells. Control indicates recipient cells stained with CTB-Biotin. MB231-CM, B3GALT4-CM, and shB3GALT4-CM indicate recipient cells with the corresponding CM treatment stained with CTB-Biotin. The above experiments were performed in triplicate wells and independently conducted three times. *p < 0.05, **p < 0.01, ***p < 0.001.

It has been reported that secretions derived from tumor cells can induce the EMT process in recipient cells (40). In the present study, we explored the effect of GM1 on the biological function of secretions by treating human normal mammary epithelial MCF-10A cells and breast cancer MCF-7 cells with conditioned medium (CM) from B3GALT4-overexpressed (termed as B3GALT4-CM) and -silenced (shB3GALT4-CM) MDA-MB-231 cells (**Figure 2A**). GM1 expression in those cells were examined by flow cytometry (**Figure 2B**) and immunofluorescence (**Figure S3A**). The migratory ability of MCF-10A and MCF-7 cells was enhanced by B3GALT4-CM, but suppressed by shB3GALT4-CM treatment (**Figures 2C, D**). Western blot results showed an increased expression of the mesenchymal marker of fibronectin and vimentin, and decreased expression of E-cadherin in B3GALT4-CM-treated MCF-10A and MCF-7 cells, and shB3GALT4-CM-treated cells displayed opposite changes in EMT marker expression (**Figures 2E, F**).

We speculated that GM1 might be transferred into recipient cells through secretions; thus, levels of GM1 in recipient cells were detected by flow cytometry. Compared to the treatment of CM from MDA-MB-231 cells (termed as MB231-CM), levels of GM1 in recipient cells were significantly enhanced by B3GALT4-CM, but decreased by shB3GALT4-CM treatment (**Figures 2G, H**). Taken together, these results demonstrated that phenotypes of recipient cells could be influenced by secretions from B3GALT4-overexpressed and -silenced MDA-MB-231 cells.

### Effects of GM1 on sEV Secretion and Internalization

**Figure 3:**
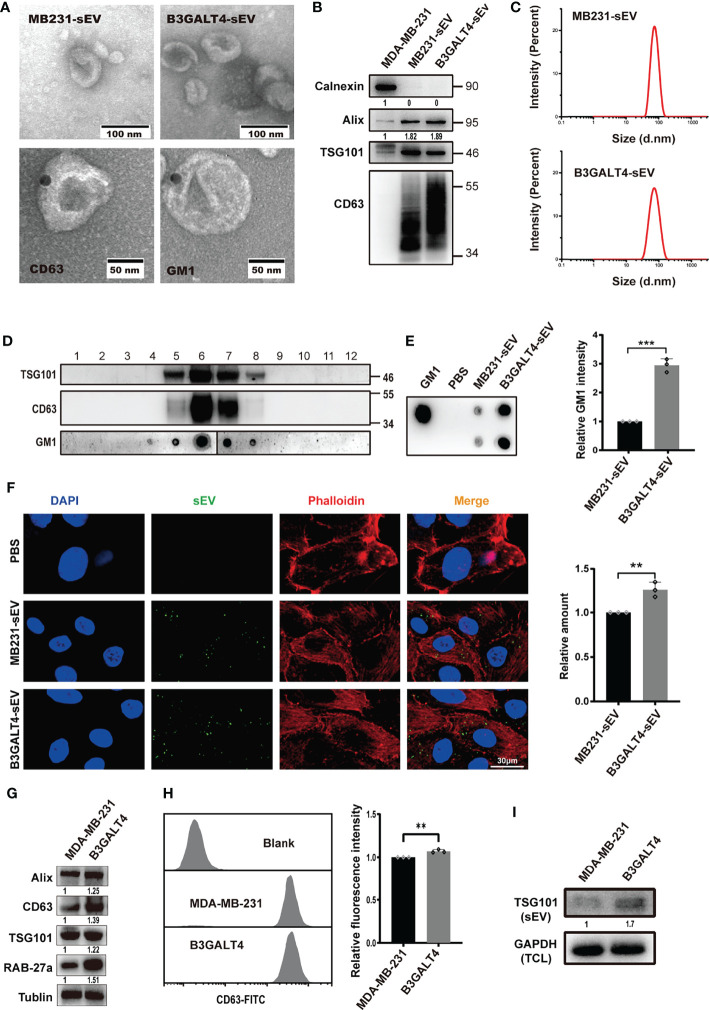
Effects of GM1 on sEV secretion and internalization. **(A)** Morphology of sEV, Immunogold-labeled CD63 on MB231-sEV and GM1 on B3GALT4-sEV evaluated by TEM. **(B)** Expression of sEV markers in MB231-sEV and B3GALT4-sEV. **(C)** Size distribution of MB231-sEV and B3GALT4-sEV evaluated by NTA. **(D)** The expression of GM1 and sEV markers in density gradient fractionation of MB231-sEV. **(E)** The expression of GM1 in MB231-sEV and B3GALT4-sEV evaluated by dot blot. **(F)** Confocal analysis of sEV uptake in MCF-10A. Signals are indicated from a merge image of CFSE-labeled sEV (green), F-actin (red), and nucleus (blue) in MCF-10A treated with MB231-sEV and B3GALT4-sEV. **(G)** Expression of sEV markers and Rab27a in cell lysates from parental and B3GALT4-transfected MDA-MB-231 cells, evaluated by Western blot. **(H)** The expression of CD63 on MDA-MB-231 cell surface, evaluated by flow cytometry. Blank indicates unstained MDA-MB-231 cells. MDA-MB-231 and B3GALT4 indicate parental and B3GALT4-overexpressed MDA-MB-231 cells stained with antibody against CD63. **(I)** Expression of sEV marker TSG101 in sEV from equal numbers of B3GALT4-overexpressed and parental MDA-MB-231 cells. **p < 0.01, ***p < 0.001.

sEV could mediate exchange of biomolecules derived from parental cells, which regulated biological characteristics of recipient cells (8). GM1 was documented to be located in MSC-sEV (41), and we determined here if GM1 was presented on sEV derived from breast cancer cells. sEV from parental or B3GALT4-overexpressed or -silenced MDA-MB-231 cells (respectively termed MB231-sEV, B3GALT4-sEV, and shB3GALT4-sEV) was isolated by a well-established differential centrifugation method. They both displayed the typical cup-shaped morphology visualized by an electron microscope (**Figures 3A**, **S3B**) and clear expression of sEV markers (CD63, Alix, and TSG101) (**Figures 3B**, **S3C**). Nanoparticle Tracking Analysis (NTA) showed that there were no significant differences in the size distribution of two kinds of sEV, which both exhibited a diameter of 30–100 nm (**Figures 3C**, **S3D**).

Density gradient centrifugation showed that GM1 was presented in CD63- and TSG101-positive fractions (**Figure 3D**), which were primarily located on sEV by immunoelectron microscopy (**Figure 3A**). GM1 levels were significantly higher in B3GALT4-sEV than MB231-sEV by dot blot analysis (**Figure 3E**). sEV was labeled with CFSE and fed to MCF-10A cells for measurement of cellular uptake, and we found that they were both internalized (**Figure 3F**). Notably, sEV from B3GALT4-overexpressed MDA-MB-231 cells was more efficiently internalized by recipient cells (**Figure 3F**). However, B3GALT4 silence suppressed sEV internalization (**Figure S3E**), indicating that sEV uptake might be determined by GM1 levels on sEV. Vesicular GM1 labeled with CTB-Biotin (**Figure S3F**) was efficiently internalized by recipient cells (**Figure S3G**), demonstrating that GM1 could be transferred from donor cells to recipient cells *via* sEV. The expression of CD63 was significantly increased in B3GALT4-overexpressed cells, examined by Western blot (**Figure 3G**) and flow cytometry (**Figure 3H**). Since CD63 was documented to be associated with sEV secretion (42), these data might indicate an enhanced sEV secretion in MDA-MB-231 cells. Meanwhile, sEV was isolated from equal numbers of B3GALT4-overexpressed and parental MDA-MB-231 cells with equal amounts of GAPDH in total cell lysates. Western blot demonstrated that the expression of TSG101 in B3GALT4-sEV was significantly higher than in sEV from parental MDA-MB-231 cells (**Figure 3I**). Collectively, these results showed that GM1 was sorted onto sEV, and elevated GM1 levels might enhance the sEV secretion and facilitate sEV internalization.

### Proteomic Analysis of Parental and B3GALT4-Transfected MDA-MB-231 Cells

**Figure 4:**
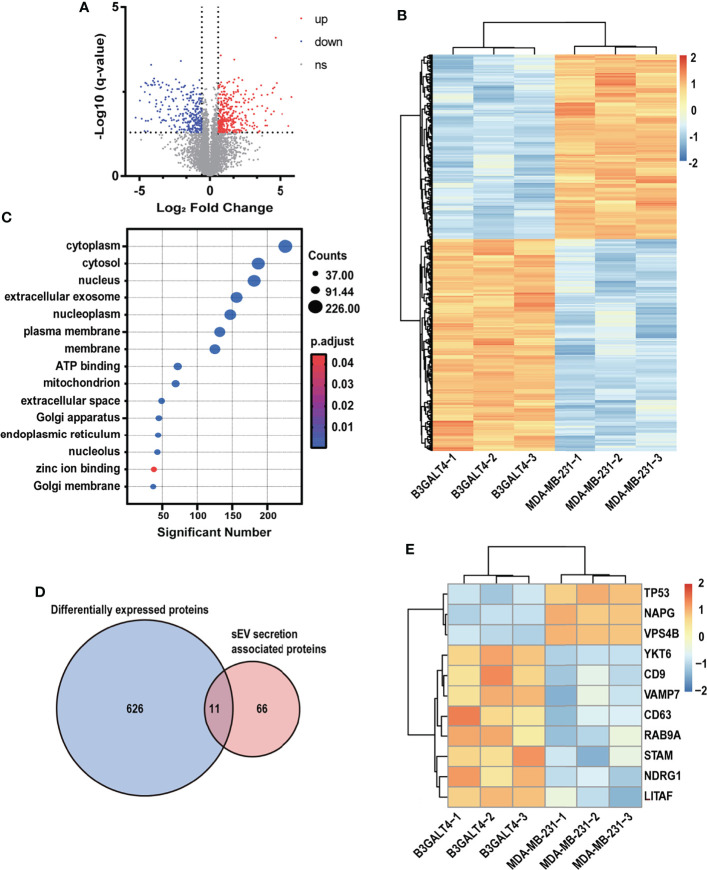
Proteomic analysis of parental and B3GALT4-transfected MDA-MB-231 cells. **(A)** Volcano plot of expression pattern of identified proteins in parental and B3GALT4-transfected MDA-MB-231 cells. The -log10 (*q*-value) is plotted against the log2 (fold change: B3GALT4-transfected cells vs. parental cells) using the cutoffs of fold change >1.5, fold change < 0.67, and *q*-value < 0.05. Red, significantly upregulated expression. Blue, significantly downregulated expression. **(B)** Expression pattern of DEPs identified in parental and B3GALT4-transfected MDA-MB-231 cells, B3GALT4-1/-2/-3 and MDA-MB-231-1/-2/-3 represent three biological replicates. Red, upregulated expression. Blue, downregulated expression. **(C)** Enriched Gene Ontology analysis of DEPs. **(D)** Venn diagram of DEPs and sEV secretion-related proteins. **(E)** Heatmap of DEPs involved in sEV secretion process. Red, upregulated expression. Blue, downregulated expression. Ns, no significant differential expression.

Heparanase was documented to enhance sEV secretion, and alter sEV cargos and functions in several human cancer cell lines (43). Our data showed that GM1 might also enhance sEV secretion in MDA-MB-231 cells (**Figures 3G–I**). To explore the effect of GM1 on sEV secretion, proteomic analysis of parental and B3GALT4-transfected MDA-MB-231 cells was performed. A total of 6,086 proteins were identified, of which 637 proteins were differentially expressed (**Table S2**). Expression patterns of dysregulated proteins in B3GALT4-transfected cells compared to parental cells were represented in a volcano plot (**Figure 4A**). A total of 341 proteins were significantly upregulated in B3GALT4-transfected cells, and 296 proteins were significantly downregulated. Correlation analysis demonstrated that the differentially expressed protein MS intensities of parental and B3GALT4-overexpressed MDA-MB-231 cells were highly correlated among biological replicates (Pearson correction factor > 0.9), suggesting that the quantification analysis had high reliability (**Figure S4A**). To illustrate the difference between the two groups, a heatmap was generated using the differentially expressed proteins (DEPs) (**Figure 4B**). Gene Ontology analysis revealed that DEPs were enriched in cell components, such as cytoplasm, nucleus, and EVs (**Figure 4C**). Our attention was drawn to proteins involved in sEV secretion, and 11 proteins were filtered by overlapping DEPs and proteins involved in the sEV secretion process (44) (**Figure 4D**). Eight proteins related to sEV secretion, namely, YKT6, CD9, VAMP7, CD63, RAB9A, STAM, NDRG1, and LITAF, were upregulated in B3GALT4-overexpressed cells, and 3 proteins, namely, TP53, NAPG, and VPS4B, were downregulated (**Figure 4E**). Proteomic analysis revealed that 11 differentially expressed sEV secretion-related proteins might be responsible for the altered sEV secretion. Furthermore, proteomic analysis of B3GALT4-overexpressed and parental MDA-MB-231 cells identified 26 DEPs defined as oncogenic proteins (45) (**Figure S4B**). Of these proteins, 15 identified oncogenic proteins were documented to be presented in sEV (46) (**Figure S4C**), and 8 upregulated oncogenic proteins were associated with the EMT process, indicating that these oncogenic proteins might be transferred to recipient cells *via* sEV. The data indicated that enhanced GM1 levels might elevate oncogenic protein levels in donor and recipient cells, and thus facilitate the EMT process.

### GM1-Enriched sEV-Induced EMT Process of Recipient Cells

**Figure 5:**
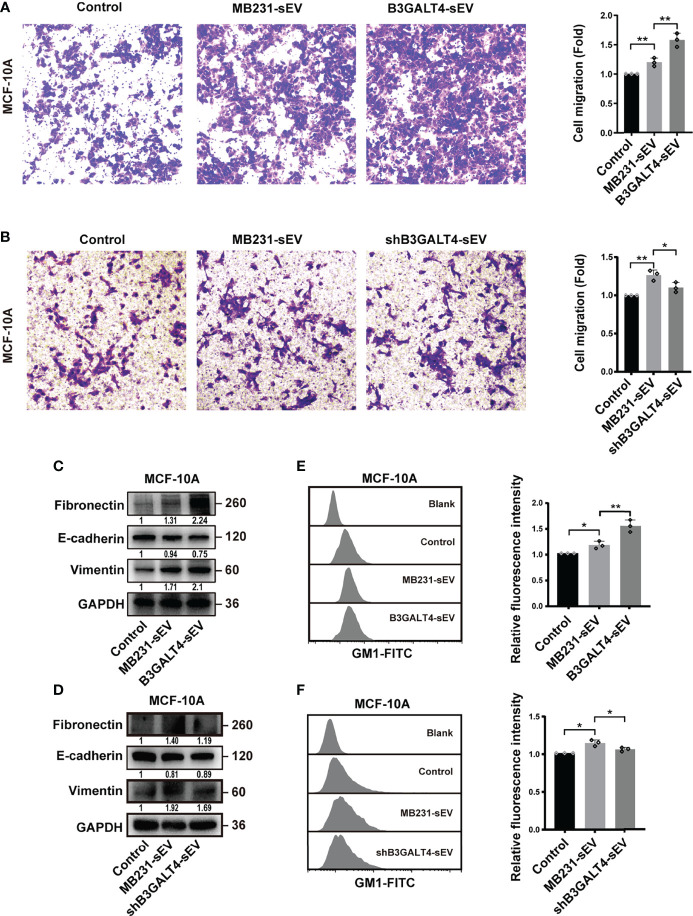
GM1-enriched sEV-induced EMT process of recipient cells. **(A, B)** The migratory ability of MCF-10A cells treated with B3GALT3-sEV **(A)** and shB3GALT4-sEV **(B)**. **(C, D)** Expression of fibronectin, E-cadherin, and vimentin in MCF-10A treated with B3GALT3-sEV **(C)** and shB3GALT4-sEV **(D)**. **(E, F)** The expression of GM1 in MCF-10A cells treated with B3GALT3-sEV **(E)** and shB3GALT4-sEV **(F)**, evaluated by flow cytometry. Blank indicates unstained MCF-10A or MCF-7 cells. Control indicates recipient cells stained with CTB-Biotin. MB231-sEV, B3GALT4-sEV, and shB3GALT4-sEV indicate recipient cells with the corresponding sEV treatment stained with CTB-Biotin. The above experiments were performed in triplicate wells and independently conducted three times. *p < 0.05, **p < 0.01.

To explore the biological function of vesicular GM1, sEV with different levels of GM1 was fed to MCF-10A cells. Compared to the control group, the migratory ability of MCF-10A cells was promoted by MB231-sEV, and further promoted by B3GALT4-sEV, but suppressed by shB3GALT4-sEV treatment (**Figures 5A, B**). Increased expression of the mesenchymal marker of fibronectin and vimentin, and decreased expression of E-cadherin were detected in B3GALT4-sEV-treated MCF-10A. shB3GALT4-sEV-treated cells displayed opposite changes in EMT marker expression (**Figures 5C, D**). The expression of GM1 in MCF-10A cells was increased after B3GALT4-sEV treatment compared to MB231-sEV treatment, but decreased by shB3GALT4-sEV treatment evaluated by flow cytometry (**Figures 5E, F**). These data indicated that vesicular GM1 could be transferred into recipient cells and further promote cell migration and induce the EMT process.

## Discussion

**Figure 6:**
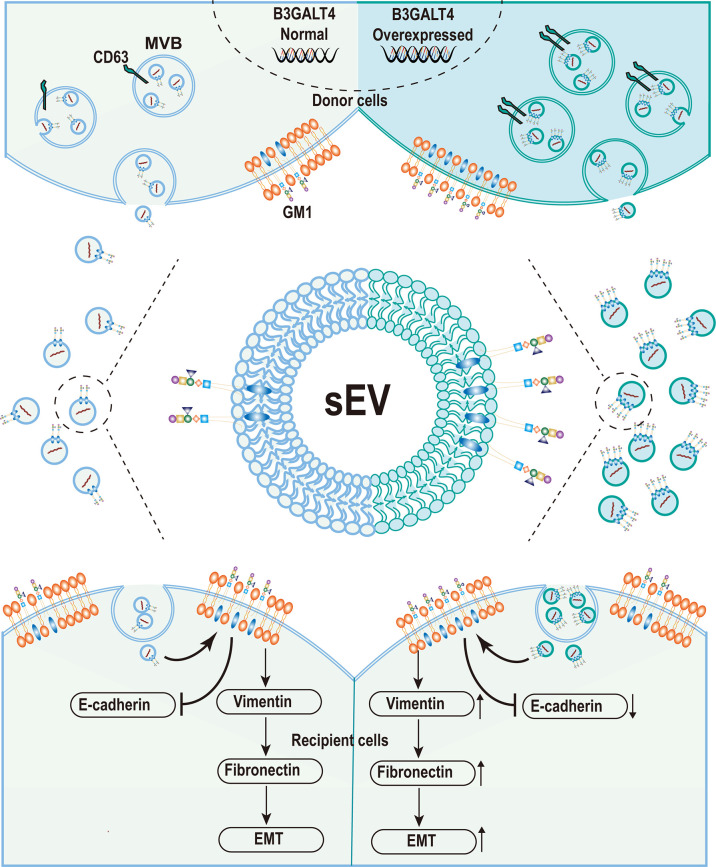
Graphical Abstract. The schematic model of ganglioside GM1 mediating the epithelial–mesenchymal process of breast epithelial cells *via* tumor-derived small extracellular vesicles.

sEV plays a pivotal regulatory role in cell–cell communication and has been implicated in many physiological processes by exchange of vesicular biomolecules including lipid, glycoconjugates, proteins, and RNAs (47, 48). Accumulated studies have documented that sEV was heavily glycosylated, and glycosylation has a great effect on sEV biosynthesis and function (49). Complex N-glycans were identified as determinants of protein trafficking into sEV (50), and vesicular glycans were also important for their uptake by recipient cells (51). Heparan-sulfate proteoglycan syndecan-1 mediated biogenesis through the syndecan-1-syntetin-Alix pathway (52), which was regulated by the heparanase activity (53). Glycosphingolipids, specifically GM1, were also documented to be located in MSC-derived sEV (41), while the function of vesicular GM1 was rarely studied. Our finding clearly demonstrated that higher level of GM1 enhanced the sEV secretion, and GM1 was presented on sEV derived from MDA-MB-231 cells. Vesicular GM1 facilitated the EMT process in recipient cells, accompanied by enhanced migratory ability (**Figure 6**).

GM1 is typically located in the outer leaflet of the plasma membranes, and regulates many cellular processes, such as cell polarization and movement (54), nerve protection, repairment (55), and cell proliferation (56, 57). GM1 regulates the localization of many membrane proteins in membrane microdomain and modulates the phenotype and function of a range of cell lines. For example, GM1 could control the movement of PDGFR from glycolipid-enriched microdomain (GEM) to the non-GEM microdomain, suppressed PDGF/PDGFR signaling, and reduced cell proliferation in mouse fibroblast Swiss 3T3 cells (58). MMP-9 was recruited to the GEM/rafts by reduced GM1 levels, contributing to increased invasion and high metastasis in mouse Lewis lung cancer cells (59). The CTB specifically binds to the branched pentasaccharide moiety of ganglioside GM1 (60), and CTB barely binds to other glycosphingolipids, including GM2, GM3, GD1a, GD1b, GT1b, asialo-GM1, GL4 globoside, and lactosyl ceramide (61). Therefore, CTB was used to bind and visualize GM1 on cell membrane. Our study showed that overexpression of GM1 induced the EMT process, accompanied by reduced cell–cell adhesion, enhanced cell–ECM adhesion, and migratory ability in MCF-10A cells. The apparent discrepancy might result from the fact that different membrane proteins were recruited to the GEM or non-GEM microdomain in different cell lines. We also found that several membrane proteins, including integrin α3, were upregulated in B3GALT4-overexpressed cells (**Figure S4D**). These proteins might be recruited to the GEM microdomain response to GM1 overexpression and contribute to the elevated cell–ECM adhesion and migratory ability.

Recent studies have revealed that many proteins were involved in sEV biogenesis and secretion. For example, CD63 knockout by CRISPR/Cas9 resulted in a reduction in sEV secretion in HEK293 cells (42). The transfection of LITAF (also called small integral membrane protein of the lysosome/late endosome, SIMPLE) in COS cells can increase sEV secretion, while mutations in LITAF can interfere with the formation of MVB (62). sEV secretion and production were also determined by small GTPases of the Rab family, and knockdown of five Rab proteins (Rab2b, Rab9a, Rab5a, Rab27a, and Rab27b) inhibited sEV secretion in HeLa cells (63), of which Rab9a was important for lysosomal biogenesis and late endosomal morphology (64). Our findings showed an increase in sEV secretion in B3GALT4-overexpressed cells, and proteomic analysis was performed to explore the mechanism underlying the enhanced sEV secretion. Eleven differentially expressed proteins were involved in sEV secretion, and several proteins, including CD63, LITAF, and Rab9a, were upregulated in GM1 transfectants. Thus, the role of GM1 in sEV secretion might depend on the expression regulation of proteins associated with sEV secretion. Moreover, recent studies documented the involvement of several lipids in the biogenesis and release of sEV. For example, inhibition of the formation of ceramide formation suppressed the release of sEV in Oli-neu cells (65), indicating that elevated GM1 expression might also contribute to the increase of sEV secretion.

The mechanism underlying the vesicular GM1-induced EMT process was also investigated. We found that GM1 levels were elevated by B3GALT4-CM and B3GALT4-sEV treatment in recipient cells. Endogenous and exogenous increases in GM1 levels facilitated the EMT process, indicating that vesicular GM1 was one of the regulators of the EMT process in recipient cells. Mechanistically, GM1 was reported to be able to change the distribution of many receptors in glycosphingolipid‐enriched microdomain and caveolae of the plasma membrane, and regulate the signaling activation (27). Similarly, vesicular GM1 might regulate the distribution of receptors on membrane and the activation of downstream signaling pathway, resulting in cell phenotype changes in recipient cells. Furthermore, proteomic analysis of B3GLAT4-overexpressed and parental MDA-MB-231 cells demonstrated an upregulated expression of several oncogenic proteins in B3GALT4-overexpressed cells (**Figures S4B, C**), including SMC1A, BIRC6, and FN1, which were documented to be related to the EMT process or migration. FN1 was upregulated in metastatic melanoma cells, and FN1 knockdown in metastatic tumor cells suppressed the EMT process, migration, invasion, and adhesion (66), and Deng et al. reported that FN1-enriched EVs derived from breast tumor cells enhanced tumor cell invasion *in vitro* and *in vivo* (67). Meanwhile, 15 DEPs of oncogenic proteins were reported to be presented in sEV by the ExoCarta dataset (46) (**Figure S4C**), indicating that these identified oncogenic proteins might be transferred into recipient cells *via* sEV. Similarly, ceramide, the key component of GSLs, is responsible for the sEV secretion and cargo loading into sEV (68). These results suggested that GM1 might modulate the oncogenic loading into sEV in a ceramide-like manner. Effects of GM1 on cargo loading into sEV and the molecular mechanism need to be explored in our future studies. These results demonstrated that B3GALT4-sEV treatment could result in an increased level of oncogenic proteins in recipient cells, and thus facilitate the EMT process. Collectively, vesicular GM1 might facilitate the EMT process through transfer of GM1 and oncogenic proteins.

In conclusion, GM1 was found to be presented on sEV derived from MDA-MB-231 cells. High level of GM1 enhanced the sEV secretion, which might result from the elevated expression of proteins related to sEV secretion in B3GALT4-overexpressed cells. Furthermore, vesicular GM1 could induce the EMT process of recipient cells, which might result from the transfer of GM1 and oncogenic proteins. Our findings might provide essential information for a deeper insight into the biological function of GSLs.

## Data Availability Statement

The original contributions presented in the study are included in the article/**Supplementary Material**. Further inquiries can be directed to the corresponding authors.

## Author Contributions

QM: Methodology, Investigation, Data curation, and Writing–original draft. DZ: Methodology, Investigation, and Writing—review and editing. FG: Supervision. XL: Investigation. XY: Methodology and Investigation. ZT: Investigation and Writing—review and editing. All authors contributed to the article and approved the submitted version.

## Funding

This study was supported by the National Science Foundation of China (Nos. 31971211 and 81802654), the Science Foundation for Distinguished Young Scholars of Shaanxi Province (2021JC-39), the Natural Science Foundation of Shaanxi Province (2019JZ-22 and 2021SF-294), and the Youth Innovation Team of Shaanxi Universities.

## Conflict of Interest

The authors declare that the research was conducted in the absence of any commercial or financial relationships that could be construed as a potential conflict of interest.

## Publisher’s Note

All claims expressed in this article are solely those of the authors and do not necessarily represent those of their affiliated organizations, or those of the publisher, the editors and the reviewers. Any product that may be evaluated in this article, or claim that may be made by its manufacturer, is not guaranteed or endorsed by the publisher.
