# Supplementary material for: Vesicular Ganglioside GM1 From Breast Tumor Cells Stimulated Epithelial-to-Mesenchymal Transition of Recipient MCF-10A Cells
Source: Front Oncol. 2022 Apr 26;12:837930. doi: 10.3389/fonc.2022.837930 (PMC9086854; doi:10.3389/fonc.2022.837930)
Supplement: Supplementary file 1 [file DataSheet_1.docx]

Supplementary Material

# Supplementary Figure S1. Dysregulation of GM1 in breast cancer and effects of GM1 on the EMT process in MCF-10A cells.

# Supplementary Figure S2. The effect of GM1 on TGF-β induced EMT process.

# Supplementary Figure S3. Characteristics of sEV from B3GALT4 silenced MDA-MB-231 cells and vesicular GM1 delivery.

# Supplementary Figure S4. DEPs identified in B3GALT4 overexpressed and parental MDA-MB-231 cells.


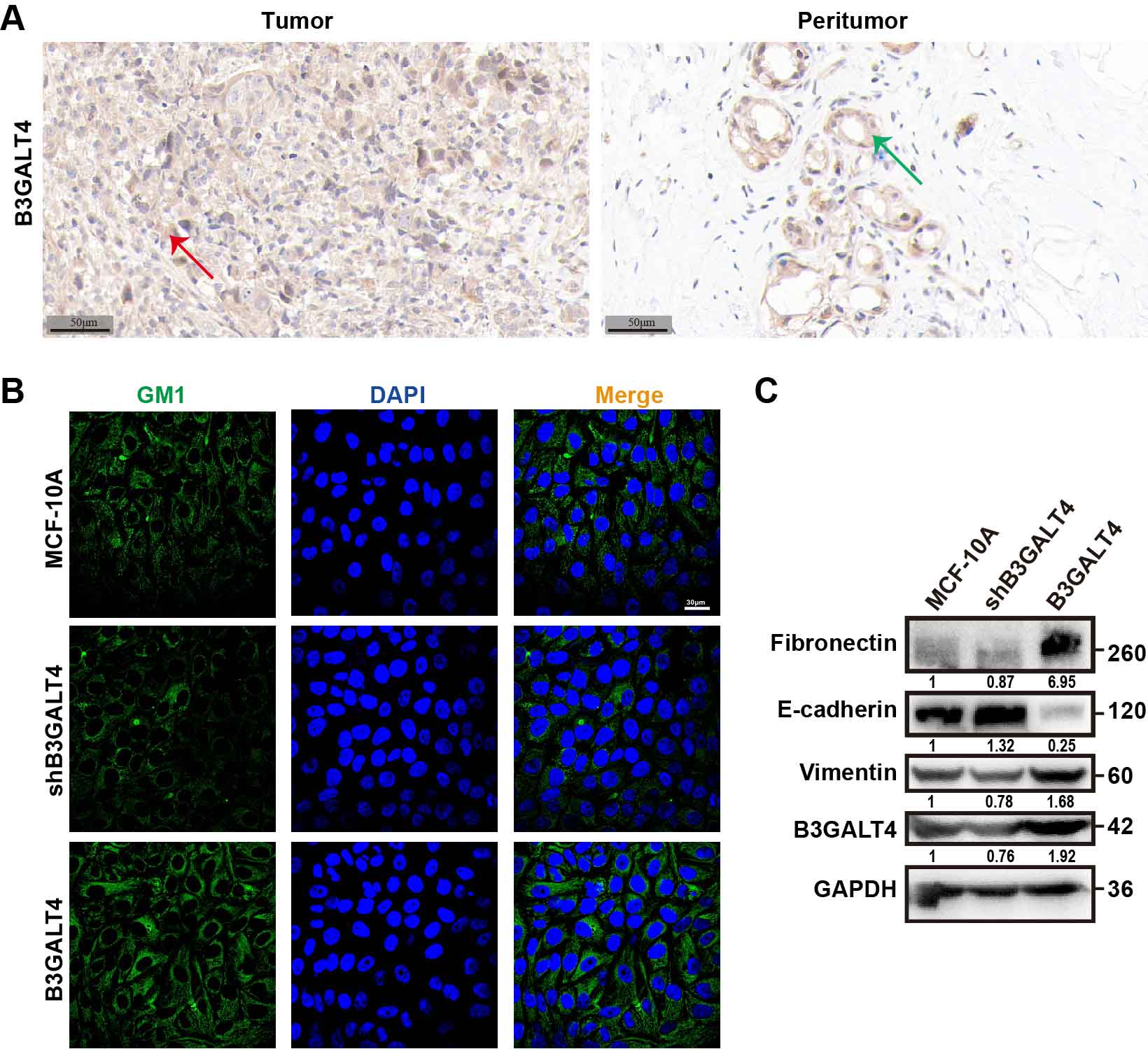


**Supplementary Figure S1. Dysregulation of GM1 in breast cancer and effects of GM1 on the EMT process in MCF-10A cells. (A)** Expression of B3GALT4 in paired breast cancer and paracancerous tissues by immunohistochemical analysis. Red arrows indicate representative positive signals in cancer cells, and green arrows indicate representative positive signals in ducts. **(B)** Immunofluorescence of GM1 in B3GALT4 overexpressed and silenced MCF-10A cells. **(C)** Expression of fibronectin, E-cadherin, vimentin and B3GALT4 in B3GALT4 overexpressed and silenced MCF-10A cells.


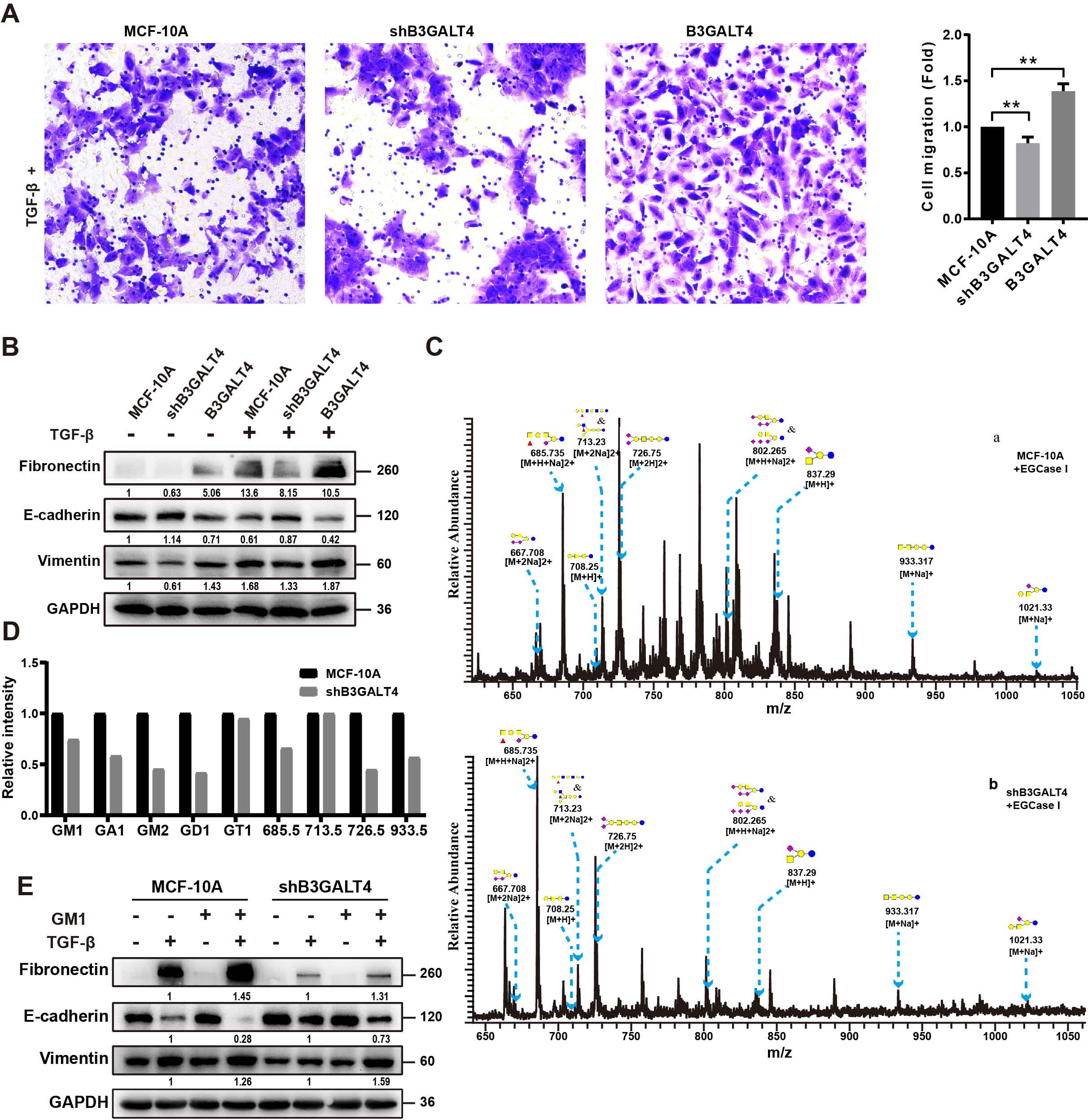


**Supplementary Figure S2. The effect of GM1 on** **TGF-β induced EMT process. (A)** The migratory ability of cells treated with TGF-β.**(B)** Expression of fibronectin, E-cadherin and vimentin in cells treated with TGF-β evaluated by western blot. **(C)** LC-MS spectra of glycan components of GSLs from parental and B3GALT4 silenced MCF-10A cells released by EGCase. **(D)** The relative intensity of identified GSLs from parental and B3GALT4 silenced MCF-10A cells. **(E)** Expression of EMT markers in cells treated with TGF-β and GM1.


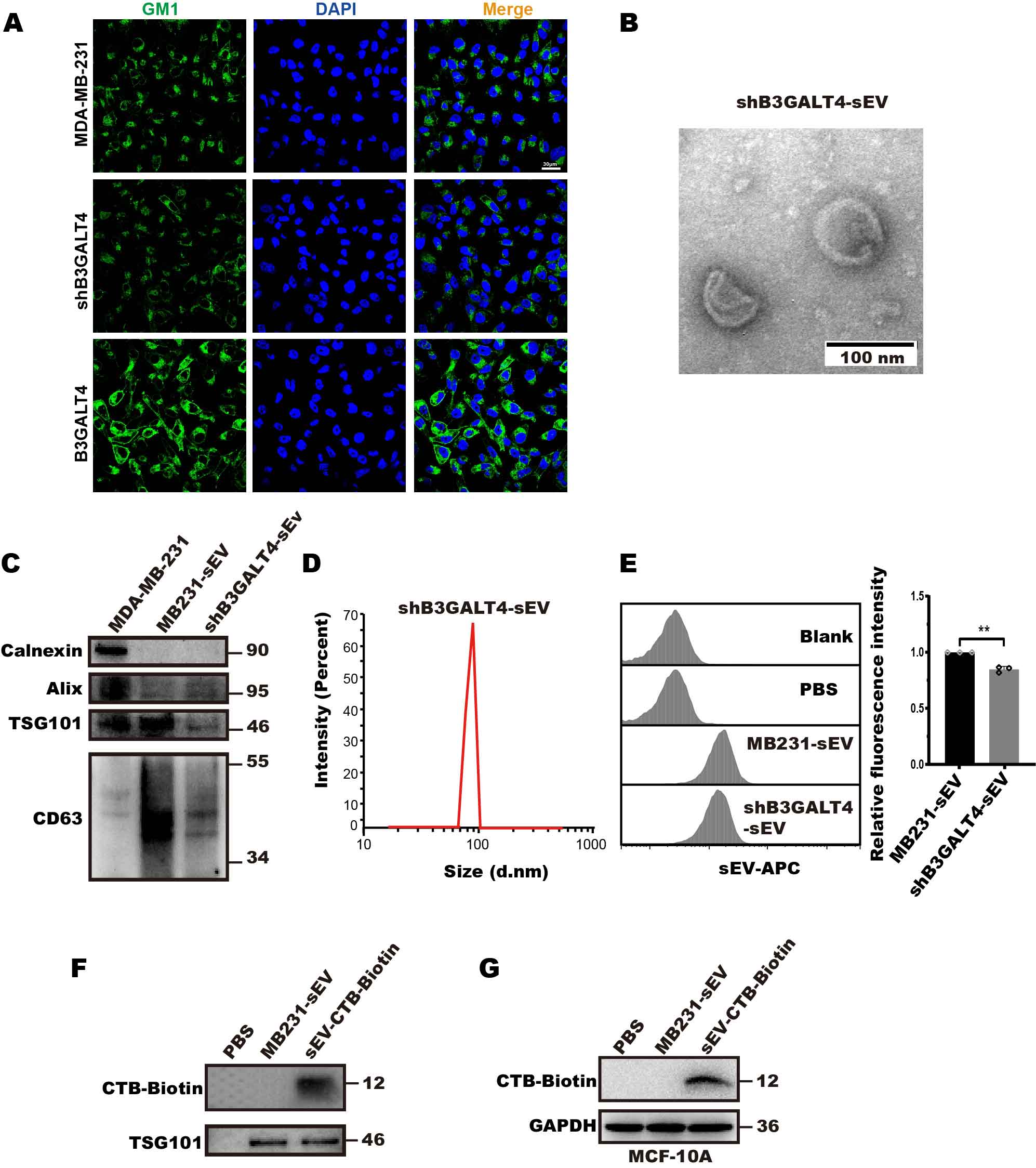


**Figure S3. Characteristics of sEV from B3GALT4 silenced MDA-MB-231 cells and vesicular GM1 delivery. (A)** Immunofluorescence of GM1 in B3GALT4 silenced and overexpressed MDA-MB-231 cells. **(B)** Morphology (evaluated by TEM) of shB3GALT4-sEV. **(C)** The expression of sEV markers in MB231-sEV and shB3GALT4-sEV. **(D)** Size distribution of shB3GALT4-sEV, evaluated by NTA. **(E)** The uptake of shB3GALT4-sEV evaluated by flow cytometry. **(F)** CTB-Biotin labeling of vesicular GM1 evaluated by western blot. **(G)** The uptake of CTB-Biotin labeled vesicular GM1 in MCF-10A cells.


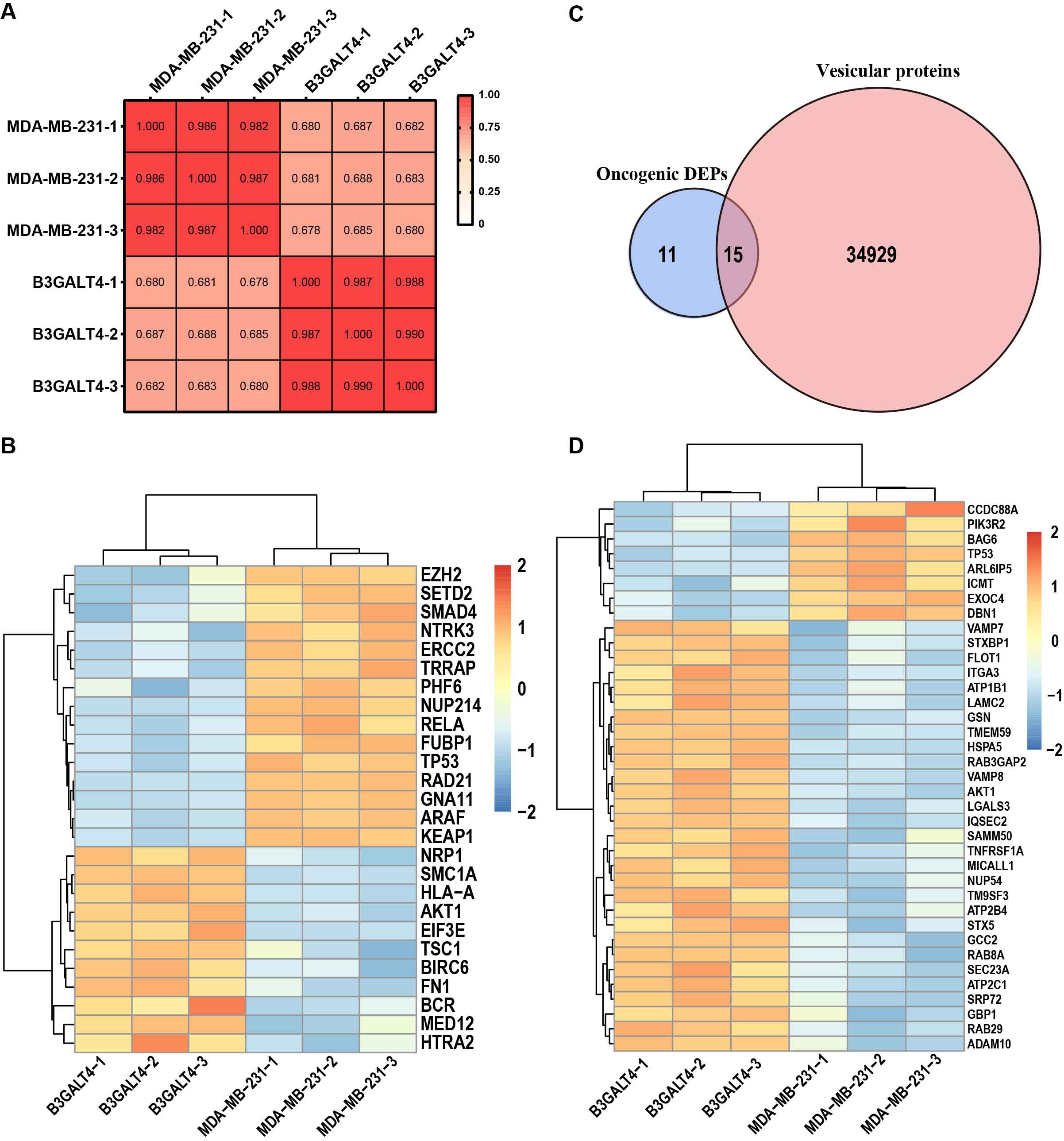


**Figure S4. DEPs identified in B3GALT4 overexpressed and parental MDA-MB-231 cells.** **(A)** Correlation analysis of the protein MS intensities of parental and B3GALT4 overexpressed MDA-MB-231 cells. **(B)** Heat map of oncogenic DEPs. Red, up-regulated expression. Blue, down-regulated expression. **(C)** Venn diagram of oncogenic DEPs and vesicular proteins from ExoCarta dataset. **(D)**Heat map of DEPs localized on membrane. Red, up-regulated expression. Blue, down-regulated expression.
