# Supplementary material for: Vesicular Ganglioside GM1 From Breast Tumor Cells Stimulated Epithelial-to-Mesenchymal Transition of Recipient MCF-10A Cells
Source: Front Oncol. 2022 Apr 26;12:837930. doi: 10.3389/fonc.2022.837930 (PMC9086854; doi:10.3389/fonc.2022.837930)
Supplement: Supplementary file 2 [file Table_1.docx]

| **Experimental (m/z)** | **Calculated (m/z)** | **Ion** | **Relative ratio**  **(10A-shB3GALT4/CON）** | **Structure** |  |
| --- | --- | --- | --- | --- | --- |
| **1021.5** | **1021.33** | **[M+Na]+** | **0.400075528** | **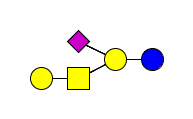** |  |
| **708.5** | **708.25** | **[M+H]+** | **0.283728646** | **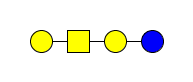** |  |
| **837.58** | **837.29** | **[M+H]+** | **0.074405824** | **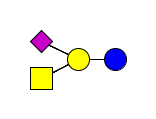** |  |
| **667.4** | **667.708** | **[M+2Na]2+** | **0.186787592** | **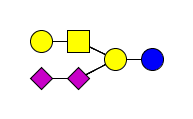** |  |
| **802.5** | **802.265** | **[M+H+Na]2+** | **0.140003191** | **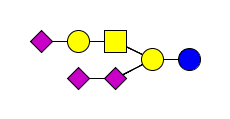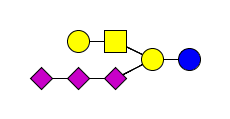** |  |
| **685.5** | **685.735** | **[M+H+Na]2+** | **0.356343998** | **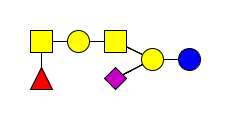** |  |
| **713.5** | **713.23** | **[M+2Na]2+** | **0.165227817** | **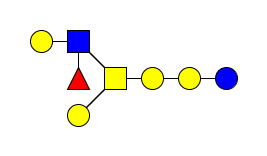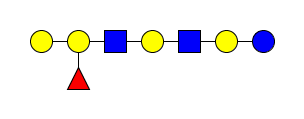** |  |
| **726.5** | **726.75** | **[M+2H]+** | **0.163560918** | **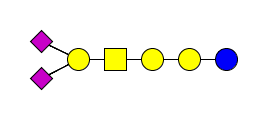** |  |
| **933.5** | **933.317** | **[M+Na]+** | **0.190330487** | **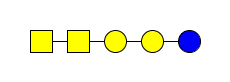** |  |

Table S1. Proposed structures and their molecular ions in mass spectra of glycan components of glycosphingolipid of parental and B3GALT4 silenced MCF-10A cells
